# Indoor Pool Game and Substance Abuse as Trajectories to Students’ Academic Procrastination: The Mediation Role of Self-Regulation

**DOI:** 10.3389/fpsyg.2022.835371

**Published:** 2022-04-18

**Authors:** Dinaol Urgessa Gita, Amanuel Tadesse Koya, Berhanu Nigussie Worku

**Affiliations:** ^1^Department of Psychology, College of Education and Behavioral Sciences, Jimma University, Jimma, Ethiopia; ^2^Department of Psychology, Institute of Social Science and Humanity, Gambella University, Gambella, Ethiopia

**Keywords:** academic procrastination, indoor pool game, mediation, substance abuse, university students

## Abstract

**Background:**

Over the last decade, indoor pool games (IPGs) and substance abuse (SA) became a remarkable emerging addictive behavior among adolescent university students. With the failure of educational quality and retention of learners, boomerangs around the university local environment in line with the students’ learning culture were not considered in many countries including Ethiopia. Thus, this study aimed to examine the trajectory and contribution of an IPG and SA to students’ academic procrastination (AP) as determinants of quality education.

**Methods:**

A sequential explanatory mixed-methods design was employed. Self-reporting questionnaires, interview guides, and an observation checklist were used to collect data. All self-reporting items were adapted from previous scales. By using simple random sampling techniques, 237 undergraduate university students were selected for obtaining the quantitative data, and using purposive sampling, 12 interviewees were selected to collect the qualitative data. The SPSS AMOS version 25 was used to compute the multiple mediation path analysis. The Hayes PROCESS macro model was used. Furthermore, the thematic content analysis method was employed for the qualitative data.

**Results:**

A direct path analysis was established between IPG, SA, and AP. The path analysis model indicated that IPG did not significantly predict AP. Moreover, SA significantly predicted AP. In addition, SR had a partial mediating effect on the relationship between IPG, SA, and AP.

**Conclusion:**

The study concluded that IPG and substances available around the university local environment found trajectories to students’ AP, which in turn affects the quality of education.

## Introduction

In meeting the demand of a rapidly changing world, every country needs a well-educated citizen that has competencies to upgrade and form a decisive alignment to future social development (United Nations Educational, Scientific and Cultural Organization [UNESCO], 1998, 2015a, 2015b; Educational, Scientific and Cultural Organization [UNESCO], 2017; American Association of Colleges of Teacher Education [AACTE], 2010; Granados-Sánchez, 2011; Kromydas, 2017). This can be achieved through the quality culture of higher education that preserves the societal standards of welfare (Vilcea, 2014; Sattler and Sonntag, 2018) and an interactive and mutually supportive relationship that helps learners progress toward their valued goals (Organization for Economic Co-operation and Development [OECD], 2019).

Moreover, to produce learners that best fit the growing demand of the 21st century, it is vital to redesign a system that aligns with educational goals and sustainable development (Scott, 2015); and it needs to be supported by different contributors and educational partners (Idrisa et al., 2012; Gross et al., 2015; Gurlui, 2015; Chantia, 2017; Chakraborty et al., 2018; O’Connor and Daniello, 2019; Pandey, 2020; Levitt, n.d.). Equally important, the university’s local environment may have roles in capitalizing on learner’s retention and academic performance. A university’s local environment is contextually defined as the social and physical availability of factors contributing to the students’ academic procrastination (AP).

In addition, educational institutions strong relationship with the educational ecosystem and this ecosystem in turn (pedagogic dimension, schools, homes, and localities) are a protective factor that enables the learners to reach their full potential (Ellis and Goodyear, 2019). Moreover, success in the education system and learners’ holistic well-being is secreted in both the university’s contribution to the community’s demand and the community’s partnership in education (Puckett, 2017).

In support of this, previous studies revealed that a conducive and supportive school environment has a vital role in the engagement of learners and learning outcomes (Cohen et al., 2009; Puckett, 2017). Thus, a mutual understanding of the goal of education between the university and external bodies, such as locally authorized professionals, communities, business owners, and voluntary groups, has a chief contribution to the positive stimulation of effective education. Having a common goals and purposes of education among the contributors put grace in the educational destination (The University of Edinburgh, 2017).

On the other hand, when educational contributors and stakeholders have different corners of concentration, failure of academic wisdom is possible. For instance, Hossein Mardi and Hossein (2015) revealed that the university students’ academic culture is largely driven by the social environment, featuring a longstanding culture of alcohol, khat, hashish, and behavioral addiction. Promotions, sponsorships of concerts, and the marketing of alcohol and other substances on and near campuses add immediacy to the pressure of the popular culture of consumption (Substance Abuse and Mental Health Services Administration, 2019).

Similarly, gambling practice is widely taking place around universities, targeting university students. Many studies reported that as governments legalized gambling and substance use as entertainment, they have become public health concerns, especially for the youth (LaPlante et al., 2009; Afifi et al., 2010; Abbott, 2017; Binde et al., 2017; Calado et al., 2017; Oksanen et al., 2019; Substance Abuse and Mental Health Services Administration, 2019; Lelonek-Kuleta et al., 2020; Mazar et al., 2020). The availability of indoor pool games (IPG; Welte et al., 2009; Tariku et al., 2015; Mehari and Koye, 2019; Anyanwu et al., 2020), personal level factors (self-esteem, false perceptions, and drug abuse), social factors (peer influence and modeling), and environmental influences (availability of gambling places and advertisements) has a strong association with gambling ramifications, such as problematic emotional and mental well-being, fragile relationship pattern, economic instability such as indebtedness, loss of savings, and assets (Tariku et al., 2015), and the wastage of time economy revealed in the form of AP for the student population (Nordby et al., 2019; Akinci, 2021).

Furthermore, Walters (2004) reported that the impact of drug tradition has effectively stayed on campuses from coast to coast. Another in-depth qualitative study conducted by Tekalign (2012) on children and the youth in Arba Minch city of Ethiopia also confirmed that drug houses around schools are one of the various factors that affect children’s and youth’s positive development. Following the wide availability of substances in the university local environment, the self-regulation (SR) (Senécal et al., 1995; Bernardo et al., 2019; Katana et al., 2019; Natashia, 2020; Valenzuela et al., 2020) and AP of learners (Park and Sperling, 2011; Yi-Chun et al., 2017; AbdiZarrin et al., 2020; Najma and Sultan, 2021; Solyst et al., 2021; Yang, 2021) are factors that need due consideration. In this regard, even though the notions of gambling and AP are emerging as topics of studies in recent decades, these studies focused on their prevalence. For instance, an in-depth phenomenological study conducted with six participants selected using snow-ball sampling in Bahir Dar city of Ethiopia indicated that a pool game is one of the most common types of problematic gambling activities (Mehari and Koye, 2019). Similarly, Getu (2018) assured that pool games were the most prevalent form of gambling that was practiced by adolescent students. Moreover, a study conducted by Tariku et al. (2015) on students (*N* = 422) ranging from 12 to 21 years of age stated that the pool game was one of the gambling activities played for money in Ethiopia. Thus, despite taking a glance at the availability of this game in the school environment, none of the previous studies clearly presented its contribution to the students’ learning from an AP aspect.

Moreover, substance abuse (SA) among college students is a growing social and health problem. A study conducted among European University students indicated that about 73% of the study participants took alcohol alone or together with cannabis/hashish and/or other illicit drugs (Colomer-Pérez et al., 2019). SA has also a relationship with AP (Wormington et al., 2012; Moes, 2016; Westgate et al., 2017; Ahmadi et al., 2020). In addition, a cross-sectional survey of a stratified random sample of 4,734 high school students aged 12–23 years in Hong Kong has found out that gambling significantly correlated with a high occurrence of alcohol and substance use (Cheung, 2014). However, to our knowledge, the contributions of an IPG and SA on the AP of university students have never been addressed, especially in similar contexts of the study area.

In the context of this study, as education institutions, specifically colleges and universities, have become more accessible, significant problems that affect the future of education appear together. In this case, no matter how the universities’ local communities support the learners and the universities’ teaching-learning processes, physiologically and psychologically addictive substances (khat and IPG- as an emerging sort of recreation) in recent days could have indirect boomerang effects on students’ learning through AP. Many previous studies focused on students’ SA. Nevertheless, these studies have not considered the mediating role of the learner’s SR in the relationship between IPG, SA, and AP of university students. To address this gap, we tried to check whether SR has a mediating role in the relationship between the above-mentioned study variables. Accordingly, the following research questions were forwarded:

1. To what extents do IPG and SA contributed to the students’ AP?
2. Does SR mediate the contribution of an IPG and SA to the university students’ AP?

## Materials and Methods

### Research Design

The study employed a sequential explanatory mixed methods design. Quantitative research was mainly the leading research approach. A qualitative approach was employed to support the quantitative part and was used in the mediation studies to identify the possible mediators and explain the causative mechanisms and the contextual factors in which they function (Behrens and Smith, 1996; Davidson, 2000; MacKinnon, 2008). The qualitative mediation study is poorly documented (Bate et al., 2012). We, therefore, gathered more in-depth information on the causal processes that can also complement the quantitative findings.

### Population and Sampling

The population of the present study was university students. A total of 507 students were randomly selected at game zones and a mini-house where they play pool games. Of the total 507 selected university students, 394 university students who had both substance use and IPG practice were identified. A total of 237 students were randomly selected from those who had a practice of both substance use and IPG. Perhaps the most important advantage of choosing a random sample is that researchers can rely on the assumptions of statistical theory to conclude from what is observed (Moore and McCabe, 2003). In the simplest random sample, all units in the population are equally likely to be selected. As a result, researchers are convinced to use simple random sampling from those who had an experience of both SA and IPG. Moreover, a total of 12 participants, five from the students and seven from local business owners, were selected purposively for the qualitative study. Students who had a practice of either SA or IPG were excluded from the study.

### Tools of Data Collection

Overusing or using a substance in a manner other than it is intended to use is a sign of SA. Taking substances more often than required or in higher doses is a sign of SA (Parikh, 2021). Moreover, Breshears et al. (2009) reported that substance use can be considered abuse when its recurrence results in a failure to fulfill obligations at work, home, or school. Similar to these findings, this study was treated in the circumstance when university students use substances or engage in IPG more often or in a manner that puts them at academic, social, psychological, and any other negative costs that make them disadvantageous.

Thus, data were collected using an adapted self-reporting questionnaire. A European School Survey Project on Alcohol and Other Drugs (European School Survey Project on Alcohol and Other Drugs, 2015) five-point alternative Likert scale ranging from “Never” = 1 to “Always” = 5 was adapted to assess the SA and IPG. For example, “When I am not playing a pool, I often think about it, ‘I play a pool longer than originally planned”’ were few sample questions used for an IPG.

The academic procrastination questionnaire was adapted from Yockey’s (2016) short-scale five-point alternative Likert scale ranging from “Strongly disagree” = 1 to “Strongly Agree” = 5, whereas SR items were adapted from Chen and Lin (2018). Few of these questions were, “I put off projects (assignments, home works, studying) until the last minute”; “I know I should work on schoolwork, but I just don’t do it” were examples of questions used for AP. These questionnaires were translated to two local languages (Afaan Oromo and Amharic languages) in addition to the English language. Forward-backward translation was done and expert evaluation was made after the questionnaire was translated to the two local languages. Semi-structured items and observation checklists were also rigorously evaluated by experts.

### Data Analysis

A pilot study was conducted with 33 sampled individuals. Pilot study participants were sampled using simple random sampling from Jimma University Institute of Technology and were not included in the main research. At alpha reliability coefficients of the pilot study participants α = 0.871 for IPG, α = 0.721 for SA, α = 0.745 for SR, and α = 0.741 for AP, the final administration of the questionnaire was done. Throughout the study, a 95% CI and a 5% margin of error were used. Mediation analysis assumptions were checked and all assumptions were met. The data were computed with the Statistical Package of Social Science (SPSS, AMOS, version 25, United States). The contribution of SA and IPG to AP through SR was examined using the Structural Equation Model (SEM) path analysis. Moreover, the qualitative data obtained through interviews were transcribed and coded by the researchers and data collection assistants. Referring to Allen (1998) that stated (i) the core category which all others events related to, (ii) the processes leading to the core category that guide the action to the core category, and (iii) the causal conditions that allude to the occurrences that lead to the development of the marvel which is the main category were applied, and a content analysis was done. Thus, the theme categorizes transcribed data into a core category, process, and causal conditions.

Ethical considerations got due attention since the study was conducted on humans and community wisdom. All ethical, legal, regulatory norms, and standards that care for human subjects were also considered. After, the rigorous evaluation and the comments of Institutional Review Boards (IRB) of the College of Education and Behavioral Sciences, a letter to conduct the study was taken from the Jimma University College of Education and the Behavioral Science Research and Post Graduate Coordinating Office after careful evaluation and comment on the research protocol. The privacy and confidentiality of the respondents were ensured. Informed consent was taken from participants.

## Results

### Socio-Demographic Variables of the Study

The mean age of the participants was 22.87 (SD = 1.79). The minimum age was 17, while the maximum was 30 years of age. Regarding the gender of the participants, 231 (97.5%) were male, whereas 6 (2.5%) were female. This indicates that a large number of the participants were male (see Table 1).

**TABLE 1:** Demographic characteristics of the participants.

Variables	
**Age (in years)**	
Mean	22.87
Standard deviation	1.79
Minimum	17
Maximum	30
**Gender**	
Male	231 (97.5%)
Female	6 (2.5%)

### Regression Analysis Predicting Academic Procrastination

**FIGURE 1:**
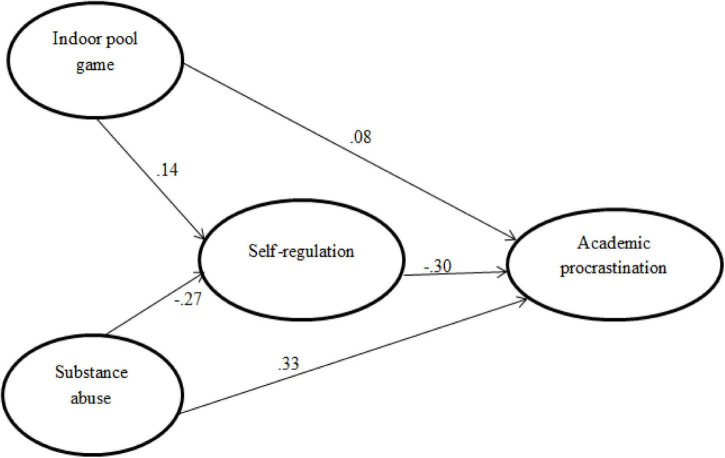
The conceptual framework of the mediation role of self-regulation in the relationship between indoor pool game, substance abuse, and academic procrastination.

As revealed in Table 2, in the first stage (Regression Equation 1), a direct path (H1) was established between IPG and AP and SA and AP as the first condition of the mediation analysis. The independent variable (IPG) did not predict the dependent variable (AP) significantly (β = 0.08, *t* = 0.554, *p* = 0.260). In the second stage (Regression Equation 2), another predictor (SA) was significantly and positively predicted by AP (β = 0.33, *t* = 5.941, *p* < 0.05). According to these findings, H2 was confirmed and 24.6% of the variance AP was explained by substance abuse. As Table 3 indicates, in the third stage (Regression Equation 3), after the inclusion of the mediator variable (SR) in the model, IPG and SA predicted AP significantly but there was a decrease in the impact coefficient of SA and a change in the direction (β = −0.283, *t* = −4.929, *p* < 0.001). According to these findings, SR had a partial mediating effect on the relationship between IPG, SA, and AP. Mediator variable analysis is shown in Figure 1 below.

**TABLE 2:** Hierarchical regression analysis predicting academic procrastination.

Reg. equal	Dependent variable	Independent variables	*B*	β	*t*	Adjusted *R*^2^	*F*
1	Academic procrastinate	In-door PG	0.041	0.08	0.554	0.246	681.023
		Subs. Abuse	0.196	0.33	5.941		
		Self-Reg.	−0.126	−0.283	−4.929		

*B, Beta; β, standardized beta; t, t-score; R^2^, Coefficient of determination; F, critical value, P < 0.05.*

**TABLE 3:** The direct and indirect path effect for the mediation model (*N* = 237).

	Model paths	Estimates	SPC	SE	CI		
					Lower	Upper	*p*
Standardized direct	IPG→AP	0.081	0.08	0.072	−0.049	0.241	0.260
	IPG→SR	0.141	0.14	0.087	−0.033	0.316	0.108
	SA→AP	0.332	0.33	0.061	0.202	0.467	0.010
	SA→SR	−0.268	−0.27	0.068	−0.429	−0.138	0.010
Standardized indirect	IPG + SA→SR→AP		−0.30	0.072	−0.109	0.009	0.010

*IPG, Indoor Pool Game; AP, Academic Procrastination; SA, Substance Abuse; SR, Self-Regulation; SPC, Standardized Path Coefficient; SE, Standard Error; CI, confidence interval.*

In Figure 1, above the indirect effect of IPG and SA on students’ AP through SR [0.14 (−27) × 0.30 = 0.123] corresponds to 26% (0.123/0.473 = 0.65) of the total effect (0.123 + 0.08 + 0.27 = 0.473). With the inclusion of the mediator variable in the model, there was a decrease of 0.143 in the variance explained in the dependent variable. When the mediator variable (SR) is not included in the model, this rate increases to 33%, and the direction changes. However, qualitative data indicate that IPG had a significant impact on the students’ academic behavior and mental well-being.

#### Interviewee 1

… *I consider gaming as a part of life. But, when I think of the time and money I spend in indoor pool gaming and gambling, I felt guilt, and hate my existence. My family hates gambling; if they know that I am gambling they surely refuse sending me the money even for copying a handout. Moreover, I couldn’t study with my full conscience after coming back from the game.*

*I have tried to stop gaming many times, yet I couldn’t do that. What is most boring in this gaming is the regression and revenge I experience after playing. The amount of money I pay for a table when I am defeated is not weigh* [*sic*] *up before and during gambling and gaming. But, if defeated, I perceive myself as I am worthless since my thought was with the money I paid followed by excessive stress, and over-anxiety to study.*

#### Another Interviewee 2

*I am a third-year economics student. The reason I go to the pool game zone is to take a rest for a while. After I start gaming if I win, I decide to play till I defeated one. If I am a winner for a long round, I perceive myself as a hero and star player. So, I never stop gaming. If I am defeated, I develop I sense of revenge that says “it is my humiliation to go until I defeat it again.” So that, I have to wait until my turn gets to and I stay for a long time. Thus, most of the time, I spent a long time at least triple of the time I planned to stay at an indoor pool game. But when I turn back, I am in intrapersonal conflicts; consider myself as I am worthless, upset, and resentful about the many I paid and the time I wasted without studying.*

#### Another Interviewee Also Suggested

*I am a psychology student at Gambella University, Ethiopia. It is not my interest to spend a long time on gaming. At the preliminary phase, whenever I plan to go to the game zones, I decide to play only two rounds. But after I get into the room unknowingly, I stay a long time. This is not a day or 2 days experience. I experience this all the time. Financial loss, academic score deterioration, and moral guilt are at the center of this practice.*

*The result from observation also revealed that participants felt restless and were eager to engage in the game. But after they played, those who were defeated in the game had intense psychological dissatisfaction behavioral and emotional disturbance, guilt feeling, and worthlessness intensively manifested after being defeated once* [*sic*] *or more rounds of in-door pool game. Wreak, pessimism, self-criticism, and emotional discomfort were also repeatedly noticed.*

On the other hand, the local business owners’ perception of IPG on the students’ academic culture was revealed as positive. Local business owners perceived game zones and IPG around the university as a means of supporting the students’ teaching-learning process.

#### Most of the Interviewees Reported

We feel as we are partners of education and also supporting the teaching-learning process. We support this through availing what makes students free whenever they want to be. In this case, indoor pool games are the main source of refreshment for university students. For instance, students who have little money which is not enough to use something at the cafeteria can play a pool game with 5 (five birr) coming to the game zone. But when they got to the cafeteria, it is inevitable to pay twofold or threefold for this. Hence, the financial cost of the encounter is not as greater as the benefit they gain.

*Moreover, students develop alternative thinking behavior and problem-solving skills. When they play a pool, they can develop self-confidence, interpersonal skills and friendship bonds, and related psychological assets as they grow to the best player. But if they go to the cafeteria, they may intake something and make communications on a certain topic(s). So, gaming has a paramount role in youths’ mental wellbeing.*

*However, most students may not come to the star player within a short period. For these individuals, the frequency of paying table, the value may be high. As a result, these individuals feel bored, angry, and also may feel guilt feeling since most of the university students are family-dependent.*

All of the effects of the path were reported. A direct path was established between IPG and AP and also between SA and AP. Of the independent variables, SA predicted the dependent variable (AP) significantly and positively (β = 0.33, *t* = 5.941, *p* = 0.010). According to these findings, 24.6% of the variance in AP was explained by SA. In the second regression equation, IPG predicted AP insignificantly (β = 0.08, *t* = 0.554, *p* = 0.260), whereas SR was accounted for IPG positively but weakly. In the third stage, after the inclusion of the mediator variable (SR) in the model, IPG and SA predicted AP significantly, but there was a decrease in the impact and change in direction of the coefficient of IPG, SA, and AP (β = −0.30, *t* = 5.982, *p* < 0.001). According to these findings, SR had a partial mediating effect on the relationship between IPG, SA, and AP.

#### Direct and Indirect Effects: The Causal Conditions, Process, and Core Category

A qualitative study obtained from the interview also revealed that IPG and SA had a direct and indirect effect on the students’ academic performance. University students reported that IPG and substance use had a reasonable effect on academic success. Participants of the study reported that they had AP which has been presented as the core category that all other categories relate to. On the other hand, a key mediating variable in this study was the students’ SR. We believed that the university students’ AP is lessened when students master SR skills based on the study variables.

As the interview transcription indicated, the ultimate aim of why students go to IPG is to restore one’s mental energy after study or to start their study with a fresh mind. As interviewees report, students who are aiming at taking a recreation after class or after study in the case of an IPG prefer the game zone. In the beginning, when they went there, they had no plan to stay in both pool game zones and substance use houses for a limited time. As a result of poor SR, students develop either a sense of inferiority and revenge when they get defeated or a sense of pride and being a star player. For instance, in IPG, if they are a winner in gaming, they are led by a sense of winning, so they never stop gaming until they get defeated.

On the other hand, if they get defeated from the very beginning, they develop a spitfire emotion, so that to clean their own moral from the inferiority complex against the winner, they counter for the second, third, or even fourth round. Even, if there are other referees, it is necessary to wait for two to three gaming rounds. After all these rounds, the defeated student starts gaming again. He waits for two-three rounds if he gets defeated again. In this case, if one round of the game takes 10 min on average, a student who waits for a game after three or four players wastes plenty of time.

……*.This is common whether it is at an examination season or not. Even, when we develop test anxiety, we think we can compensate for a consumed time at gaming in the nighttime. When we turn back to study is in this mood, we have a sense of urgency in which we get insisted to address all the contents but very few things internalized. Moreover, there is a circumstance in which we encounter relive about the game and also a nightmare when we are not there physically.*

## Discussion

Unrestrained ways of life in the university are being considered potential risk factors for the adoption of unhealthy behaviors. A self-regulatory problematic behavior manifested in the form of not starting or finishing tasks on time (Argiropoulou and Kalantzi-Azizi, 2016). A study conducted by Rabin et al. (2011) on college students (*n* = 212) affirmed that AP negatively impacted learning, achievement, academic self-efficacy, and quality of life; it was also considered a problem of executive dysfunction. A similar pattern of results was obtained in this study revealing that university students who had procrastination experience also had poor academic performance and poor psychological well-being. Moreover, a study conducted by Akinci (2021) on problematic smartphone use, SR, AP, and academic stress of 632 university students reported that AP negatively accounted for SR. Our study result is also consistent with the findings of Eissa and Khalifa (2020) and Saad (2020) who reported that SR learning and AP had a relationship.

Nowadays, many university students are spending their time on IPG either in the form of gambling or without gambling. Also, this study revealed that SA and IPG are contributing factors to the students’ AP. In this regard, in the literature, even though there is a significant lack of evidence regarding the students’ experience of IPG, this study agrees with a study conducted by Hossein Mardi and Hossein (2015) that reported university student’s academic culture is largely driven by the social environment, featuring a longstanding culture of alcohol, khat, hashish, and behavioral addiction. Moreover, a study conducted by the Molinaro et al. (2020) revealed that gaming and gambling are emerging risk behaviors with a tendency of a high degree of normalization in societies and the culture of gambling within the family environment. Similarly, the Substance Abuse and Mental Health Services Administration (2019) reported that promotion, making sponsorship of concerts, and marketing of alcohol and other substances on and near campuses add immediacy to the pressure of students’ consumption of popular culture (Substance Abuse and Mental Health Services Administration, 2019).

A cross-sectional study conducted on 250 Medical Science students also showed that students with high AP experience high difficulties in emotion regulation (Bytamar et al., 2020a,b). Moreover, experimental research revealed that the SR had the power to capitalize the learners’ ability to overcome AP (Motiea et al., 2012a,b). In our study, we found that the university students’ AP was lowered when students mastered the SR skill. In line with this, very little literature explained the relationship between alcohol use and AP (Moes, 2016; Tesfa et al., 2017; Westgate et al., 2017; Ahmadi et al., 2020). So, this is in agreement with previous studies. However, no study to date has examined the contribution of IPG to the students’ AP and also the mediation role of SR on the relationship between the study variables.

On the other hand, SR is a notion that gets consideration together with the incidence of students’ AP. As a study on a sample of 503 Chinese college students indicated, SR moderated the students’ AP and learners’ procrastination was negatively correlated with time usage character and self-control (Zhao et al., 2019). Similarly, in this study, SR mediated the students’ AP. Furthermore, procrastination behavior has an impact not only on learners’ academic achievement but also on their mental and psychological well-being (Sirois, 2007; Smolets, 2019). For instance, in the study conducted on a sample of 140 Chinese medical students (Khalid et al., 2019), procrastination induced stress among students. Muliani et al. (2020) also confirmed that there was a significant relationship between procrastination and stress. This finding is in total agreement with our study.

## Conclusion

Over the last decade, IPG and drug abuse became a remarkable emerging addictive behavior among adolescents, especially among university students as important target groups. This study disclosed that university students had IPG and drug abuse practices. There was also a reasonable positive relationship between the students’ IPG practice and AP. On the other hand, there was a moderate and positive relationship between drug abuse and AP. Conclusively, despite the levels of variation among students, IPG and drugs such as khat, alcohol, and hashish around the university’s local environment had a significant impact on the students’ AP.

### Limitations

The study attempted to identify the students’ culture of an IPG, drug use, and AP. The time which students spent may be different from individual to individual and this was not considered. Yet, we have not examined the average time they spent using illicit drugs. Moreover, this study did not identify the time they spent on gaming or gambling based on school days and non-school days.

### Implications

Despite the disclosed limitations, this study set forth inferences for practice and contributed to the existing bodies of knowledge in the area. The practical implication of this study is valuable to professionals in the field of education, psychology, sociology, social work, health, policymaking, community studies, and family studies. On this subject, since the practice of IPG, gambling, and SA culture are widely emerging incidents with adolescents at universities becoming accessible, the issue needs to get the necessary attention. Thus, the findings from this study have significant implications for the future of education and the well-being of learners.

## Data Availability Statement

The data that support the findings of this study are available from the corresponding author, upon reasonable request.

## Ethics Statement

The studies involving human participants were reviewed and approved by the Institutional Review Board of College of Education and Behavioral Sciences. The patients/participants provided their written informed consent to participate in this study.

## Author Contributions

DG conceived the study, drafted the proposal, and collected and analyzed the data. AK wrote the manuscript draft. BW contributed to proposal development and proofreading the manuscript. All authors contributed to the article and approved the submitted version.

## Conflict of Interest

The authors declare that the research was conducted in the absence of any commercial or financial relationships that could be construed as a potential conflict of interest.

## Publisher’s Note

All claims expressed in this article are solely those of the authors and do not necessarily represent those of their affiliated organizations, or those of the publisher, the editors and the reviewers. Any product that may be evaluated in this article, or claim that may be made by its manufacturer, is not guaranteed or endorsed by the publisher.
